# Preparation, Characterization and Thermo-Chromic Properties of EVA/VO_2_ Laminate Films for Smart Window Applications and Energy Efficiency in Building

**DOI:** 10.3390/ma10010053

**Published:** 2017-01-11

**Authors:** Onruthai Srirodpai, Jatuphorn Wootthikanokkhan, Saiwan Nawalertpanya, Kitti Yuwawech, Vissanu Meeyoo

**Affiliations:** 1 School of Energy, Environment and Materials, King Mongkut’s University of Technology Thonburi (KMUTT), Bangkok 10140, Thailand; nampoung.sweet@gmail.com (O.S.); kiay_kitti@hotmail.com (K.Y.); 2 Nanotec–KMUTT Center of Excellence on Hybrid Nanomaterials for Alternative Energy, King Mongkut’s University of Technology (KMUTT), Thonburi, Bangkok 10140, Thailand; saiwan.bua@kmutt.ac.th (S.N.); vissanu@mut.ac.th (V.M.); 3 Department of Chemical Engineering, Faculty of Engineering, King Mongkut’s University of Technology Thonburi (KMUTT), Bangkok 10140, Thailand; 4 Department of Chemical Engineering, Mahanakorn University of Technology, Bangkok 10530, Thailand

**Keywords:** thermochromic, VO_2_, smart windows, EVA, composite

## Abstract

Thermochromic films based on vanadium dioxide (VO_2_)/ethylene vinyl acetate copolymer (EVA) composite were developed. The monoclinic VO_2_ particles was firstly prepared via hydrothermal and calcination processes. The effects of hydrothermal time and tungsten doping agent on crystal structure and morphology of the calcined metal oxides were reported. After that, 1 wt % of the prepared VO_2_ powder was mixed with EVA compound, using two different mixing processes. It was found that mechanical properties of the EVA/VO_2_ films prepared by the melt process were superior to those of which prepared by the solution process. On the other hand, percentage visible light transmittance of the solution casted EVA/VO_2_ film was greater than that of the melt processed composite film. This was related to the different gel content of EVA rubber and state of dispersion and distribution of VO_2_ within the polymer matrix phase. Thermochromic behaviors and heat reflectance of the EVA/VO_2_ film were also verified. In overall, this study demonstrated that it was possible to develop a thermochromic film using the polymer composite approach. In this regard, the mixing condition was found to be one of the most important factors affecting morphology and thermo-mechanical properties of the films.

## 1. Introduction

It has been reported that energy use for heating and air conditioning (HVAC) accounted for 48%, 55% and 52% of buildings’ energy consumption in the USA, UK and Spain [1], respectively. To reduce the energy consumption in buildings, there has been a considerable interest in a development of so called “energy efficient windows” or “smart windows”. This effect can be achieved by several approaches including by coating chromic material onto glass substrate. In general, different types of chromic materials are available, depending on the types of external stimulus such as light (photo-chromic), heat (thermo-chromic), and electricity (electro-chromic). In this regard, thermo-chromic smart windows have received particular interest due to the fact that they can be responded to the environmental temperature and yet the visible light transparency of the thermo-chromic smart windows remains almost unchanged.

Transition metal oxides such as Ti_2_O_3_, V_2_O_3_, and VO_2_ are known to be capable of exhibiting thermo-chromic behavior. These materials are basically semi-conductors at low temperature and change to a metallic state at a temperature above its critical transition temperature. Among these metal oxides, VO_2_ has received interest and is being considered as a promising candidate for this technology. Upon heating to above its critical transition temperature (Tc, 68 °C), the material exhibits a structural change from a monoclinic to a tetragonal phase. This brings about some changes in optical and electrical properties of the material. Specifically, above Tc, the material is capable of reflecting the near infrared (NIR) light. Besides this, the transition temperature of the material can be further reduced using doping agents such as tungsten [2]. In this regard, the higher the molar percentage of dopants, the lower the transition temperature [3]. In addition, Wang et al. [4] reported that co-doping of VO_2_ with tungsten (W) and magnesium (Mg) could provide a synergistic effect in which both transition temperature and luminous transmittance of the VO_2_ film can be improved.

It is of noteworthy that, as single crystal, VO_2_ lattice cannot resist to the stress received during phase transformation and will crack after undergoing only some transition cycle. Prepared as thin film coated on selected substrate, VO_2_ film can stand more transition cycle and would be more effective for smart window application.

Progress in the developments of VO_2_ for smart thermo-chromic coatings has been recently reviewed by Wang et al. [5]. Various aspects related to the development of the materials have been discussed, including the fabrication process of VO_2_ films, strategies for improving thermo-chromic properties, and the future research directions. In terms of the fabrication processes, various methods can be used to prepare the VO_2_ thermo-chromic coating glass, including sol-gel [6], sputtering deposition [7] and chemical vapor deposition [8]. The gas phase techniques are superior in term of the precise control of process parameters and film features (thickness, microstructure). However, complex equipment is usually required. On the other hand, the sol-gel method is of low cost and feasible for metal doping. Recently, an alternative solution-based process for preparing VO_2_ thin film, namely the “polymer-assisted deposition (PAD) process” has been developed [9,10,11]. VO_2_ film with a greater transparency (40%–84%) has been claimed. This technique is interesting and might be used to fabricate smart glass at a laboratory scale. However, to fabricate larger-sized smart glass for industrial use, a different manufacturing process needs to be developed.

In this study, to avoid the above limitations, a different approach was proposed for fabricating an energy efficient window. Rather than coating thermo-chromic material onto the glass substrate, the thermo-chromic material in a powder form was directly incorporated into a polymer matrix prior to fabricating the laminated glasses. In this regard, the VO_2_ in a powder form has to be prepared. This can be done by using methods such as spray pyrolysis [12] and hydrothermal [13,14]. Chemicals used as precursor for preparing the VO_2_ include V_2_O_5_ [15,16], and NH_4_VO_3_ [17,18]. However, the synthesis of monoclinic vanadium dioxide (VO_2_(M)) via the hydrothermal process is not straight forward. This is due to the facts that vanadium oxide (VO*_x_*) comprise of up to 20 stable phases and the reaction is very sensitive to many parameters such as the calcination temperature [19] and the size and design of the reactor, which was in turn affecting the heat flow and the actual residence time. In this study, the effects of hydrothermal time and concentration of the tungsten doping agent on micro-structure of the synthesized VO*_x_* were studied and reported.

The VO_2_(M) powder has been utilized by mixing with some polymers. Shi et al. [19] for example, investigated structure-properties of glass coating, based on an acrylic polymer composite. The polymer was firstly mixed with VO_2_ via a solution process, using xylene as a solvent. Results from the Vis/NIR transmittance spectra at 15 °C and 40 °C indicate that the coating exhibited a good thermo-chromic performance. It was also found that XRD (X-ray powder diffraction) patterns and DSC (differential scanning calorimetry) thermograms of the W-doped VO_2_ changed with the size of VO_2_ particles, which was controlled by the grinding process. Similarly, Suzuki et al. [20] prepared VO_2_ coated SiO_2_ nanoparticles. The co-metal oxides were then mixed with poly(lactic acid) (PLA) using N,N-dimethylholmamid as solvent and the composite film was fabricated via a solvent casting technique. From FTIR spectra of the composite, it was found that percentage transmittance of the peaks recorded at 80 °C was lower than that of which recorded at a room temperature. This was claimed as evidence supporting the thermo-chromic behavior of the system.

As aforementioned, it is rather clear that thermo-chromic behaviors of the neat VO_2_(M) still exist once after the material has been incorporated into the polymer films. These properties are also dependent with morphology of the polymer/VO_2_ composites. This was, in turn, affected by the mixing process and the mixing conditions. In relation to our present study, the ethylene-vinyl acetate copolymer (EVA), commonly used as a binder film for the laminated glass industry, was selected as a matrix for mixing with the VO_2_ particles Normally, the commercial EVA film for either solar cell module or laminated glass is prepared via a polymer melted process such as an extrusion. In relation to this study, it is of unfortunate that a study on structure-properties of the EVA/VO_2_ film prepared by melted mixing process has been seldom reported in any open literature. In our opinion, this is an aspect deserving a consideration, taking into account that structure and properties of the EVA/VO_2_ composite prepared via a melt mixing could have been different to those of which prepared via a solution based process. Therefore, the primary aim of this work was to investigate the effect of monoclinic VO_2_ particles on heat reflectance, thermo-chromic behavior, optical transparency, and mechanical properties of the EVA based films. Comparisons on properties of the EVA/VO_2_ films prepared by two different mixing and fabrication techniques, which are a melted mixing process and a solution mixing process, were also of our interest.

## 2. Results and Discussion

### 2.1. Crystal Structures of VO_2_

**Figure 1:**
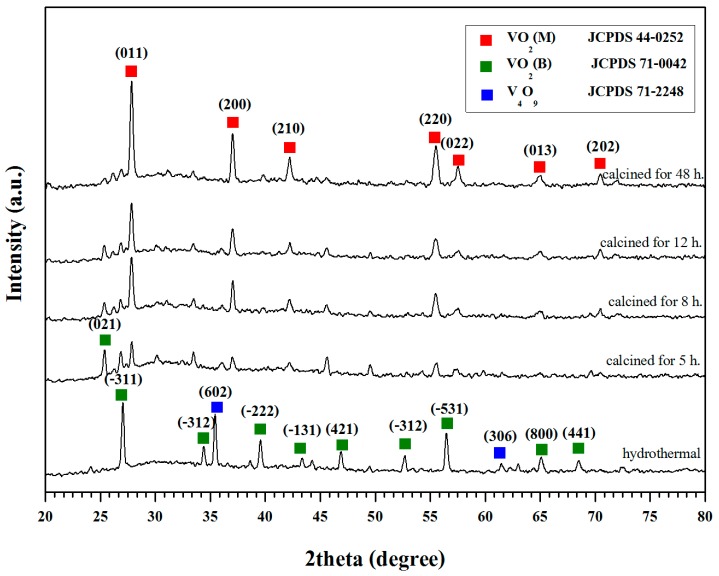
XRD patterns of the products from hydrothermal and calcination processes.

Figure 1 shows XRD patterns of the products obtained from the hydrothermal and calcination processes. The characteristic XRD peaks at 2 theta of 27.11°, 34.49°, 39.59°, and 56.33°, representing the VO_2_(B) phase, can be observed after the hydrothermal treatment. These correspond to the (−311), (−312), (−222), and (−531) planes of the metal oxide crystal. Besides, additional peaks at 35.58° and 61.13° also exist. These are attributed to the (602) and (306) plane of V_4_O_9_, which could be an intermediate product of the process (see Equations (1)–(3)). However, by further treating these materials through the calcination process, the above XRD peaks disappeared whereas those of which representing the characteristic pattern of monoclinic vanadium dioxide (VO_2_(M)) immerged. The latter include the peaks at 2θ of 27.86°, 37.05°, 42.23°, 55.53°, 57.53°, 65.00° and 70.44°, corresponding to the crystal planes of (011), (200), (210), (220), (022), (013) and (202) of VO_2_(M), respectively [17,21]. Furthermore, by analyzing the XRD peak of (011) plane with the Scherrer’s equation, crystal size of the VO_2_(M) can be calculated. The value obtained was 26.9 nm which is close to that was reported by Ji et al. (17.8 nm) [22] and Chen et al. (25 nm) [18].

2V_2_O_5_(s) + N_2_H_4_·HCl(s) + 7HCl(l) → 4VOCl_2_(aq) + N_2_(g) + 6H_2_O(g) (1)

2VOCl_2_(aq) --------hydrothermal process-----> 2VO_2_(B)(s) + 2Cl_2_(aq) (2)

VO_2_(B)(s) ----annealing process----> VO_2_(M)(s) (3)

Noteworthy, the XRD patterns significantly changes with hydrothermal time used. The XRD peak at 2θ = 27.11°, representing the VO_2_(B) intermediate was observed when the sample was treated by the hydrothermal process for about 5–12 h. However, an intensity of the above peak tended to decrease with time and eventually disappeared after treated by the hydrothermal process for 48 h. Likewise, intensity of the XRD peak representing the VO_2_(M) phase increased with time, suggesting that a sufficient time is needed for the VO_2_(B) phase to be completely converted into the VO_2_(M) phase [13,23,24]. The optimum time for achieving the completed formation of VO_2_(M) from this study is shorter than that was reported by Lv et al. [15] and Cao et al. [25]. In those cases, the hydrothermal time required to achieve a complete formation of VO_2_(M) was about 3–7 days, which is much longer than herein. In our opinion, the above discrepancies can be attributed to the different hydrothermal conditions used. A one step hydrothermal process was used in the literature work whereas two steps process was used in this study.

**Figure 2:**
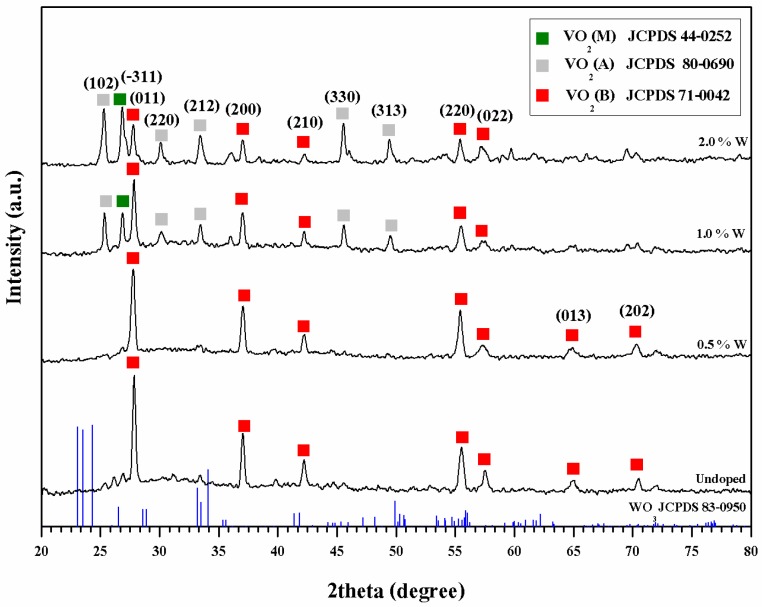
XRD patterns of VO_2_(M) and the varied tunsten doped VO_2_ (V_1−*x*_W*_x_*O_2_) obtained from the calcination process.

The similar XRD patterns were obtained when the VO_2_ was doped with 0.5 at % of tungsten (W) (see Figure 2). By further increasing the W content to 1% and 2% atom, additional peaks at 2θ of 25.47° and 27.11° which represent the meta stable tetragonal structure of VO_2_(A) and VO_2_(B) phases were also noted. The intensity values of both peaks tend to increase with the percentage atom of tungsten used. This was probably due to the differences in energy required for the formation of VO_2_(M), VO_2_(B) and VO_2_(A), which are −7.18 eV, −6.66 eV and −7.14 eV, respectively [26]. In this case, it was possible that the formation of VO_2_(A) and VO_2_(B) became more favorable, especially when the amount of tungsten used are sufficiently high. 

**Figure 3:**
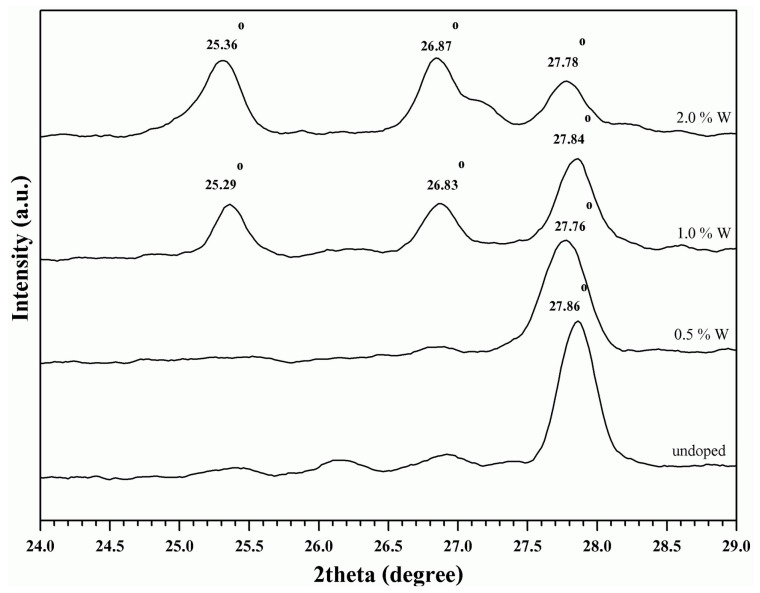
The enlarged XRD patterns (24°–29°) of VO_2_(M) and the doped metal oxides (V_1−*x*_W*_x_*O_2_) obtained by applying various concentration of the dopant (W).

From the enlarged XRD patterns (Figure 3), it was noted that the peaks, representing VO_2_(M) (2θ = 27°–29°) and those of which representing the tungsten doped VO_2_ (V_1−*x*_W*_x_*O_2_) (2θ = 24°–26° ) slightly shifted downward after doping. This can be related to an increase of inter-planar distance or d-spacing of the crystal. Since the radius of tungsten cation (W^6+^) is greater than that of the vanadium cation (V^4+^) [15,18,27], it was possible that the replacement of V^4+^ by W^6+^ in the crystal structure of VO_2_ contributed to the increase of d-spacing. In addition, by using the data from (011) plane of VO_2_ in combination with the Scherrer’s equation, the sizes of the VO_2_(M) and V_1−*x*_W*_x_*O_2_ crystal were calculated and summarized in Table 1. It was found that crystal size of the doped VO_2_ decreased as compared to that of the normal VO_2_(M). This could be attributed to the capability of W^6+^ in inhibiting growth process of the crystal. However, as the concentration of tungsten dopant was further increased above 0.5 at %, sizes of the crystals increased again. The above trend is contradicted to that was observed by Xiao et al. [27] whereby crystal size of the tungsten doped VO_2_ linearly decreased with the dopant concentration. In our opinion, the above discrepancy could be attributed to the facts that different type of reactors and calcination conditions were used. Consequently, the VO_2_(A) by-product was obtained in this study. The formation of VO_2_(A) could compete with the growth process of VO_2_. This led to the non-linear relationship between crystal size and the dopant concentration. It was worth mentioning that the above XRD pattern lacks the presence of peaks belonging to the neat tungsten oxide [18,24,28].

**Figure 4:**
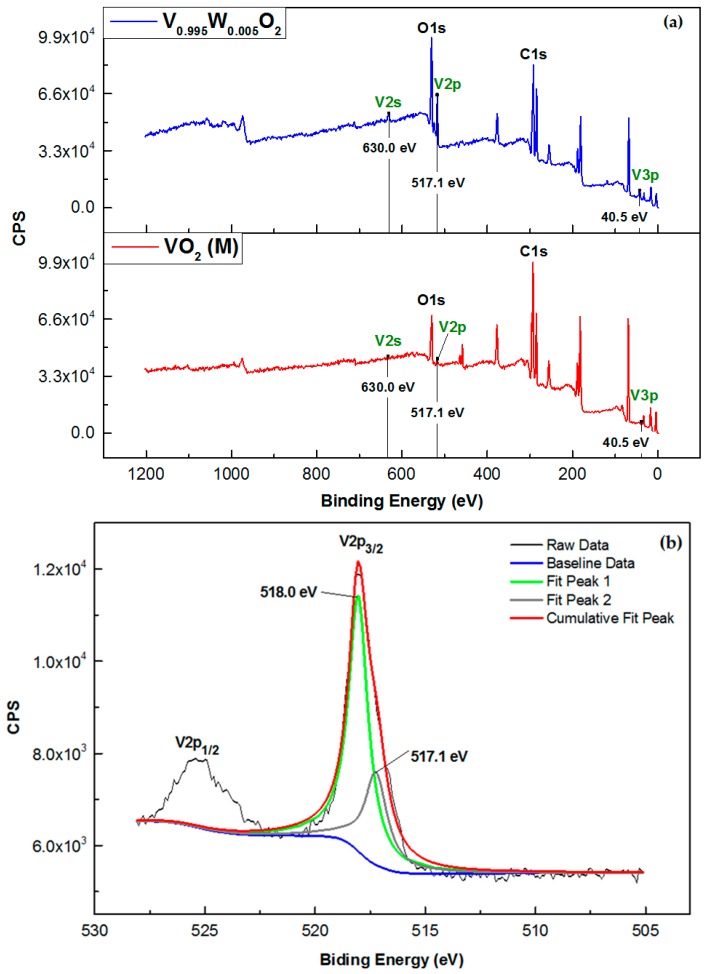
XPS survey spectra (**a**) and the high resolution or detailed spectra (**b**) of VO_2_ and the doped VO_2_ (0.5% tungsten).

The X-ray photoelectron spectroscopy (XPS) spectra of both pure and doped VO_2_ is depicted in Figure 4. The peaks representing vanadium, oxygen atoms can be noted. After doping VO_2_ with 0.5 at % of W, the characteristic peak representing the W_4f7/2_, which is normally occurs at 32.4 eV, cannot be clearly seen. This was probably due to a small amount of the dopant used. However, it was of noteworthy that an intensity of the V2p peak, representing the monoclinic phase VO_2_ increased after adding 0.5 at % W to the system. A consideration of the high resolution XPS spectra (Figure 4b) shows that the V2p peak can be separated into two peaks which are V2p_1/2_ and V2p_3/2_. Furthermore, by carrying out a deconvolution of the V2p_3/2_ peak, using the Shirley function, it was found that addition peak at 517.1 eV, representing valence state V^4+^ [29] of the doped VO_2_ (V_0.995_W_0.005_O_2_) can be noted. The binding energy of this peak is considered higher than that of the pure VO_2_(M) (515.8 eV) [22,30]. This indicates that there are 2 valence states of the vanadium after doping. This can be considered as an indirect evidence supporting the incorporation of tungsten into the VO_2_(M).

### 2.2. Morphology

**Figure 5:**
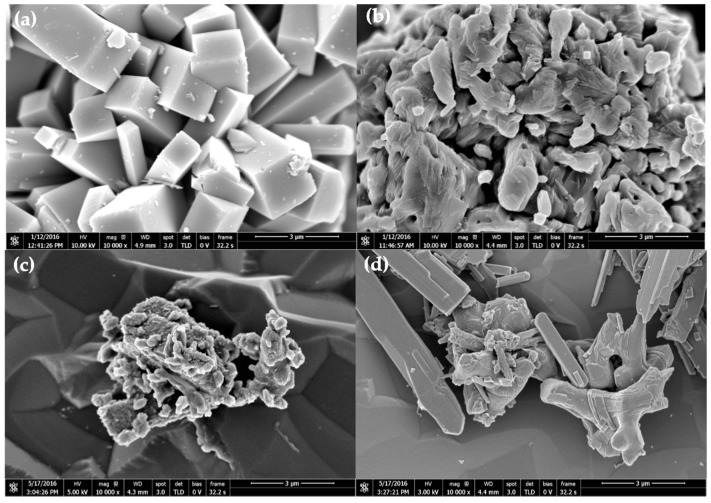
SEM images of VO_2_ obtained from hydrothermal (**a**); and calcination (**b**); and the doped VO_2_(M); V_0.995_W_0.005_O_2_ (**c**); and V_0.98_ W_0.02_O_2_ (**d**).

Figure 5 show the SEM images of the products obtained from hydrothermal and calcination processes. The tretrahedral prism shape, corresponding to the VO_2_(B) phase, was obtained after the hydrothermal. The above morphology changed to granular shape particles after calcination, some of which are being agglomerated. This corresponds to the VO_2_(M) phase. Similarly, the calcined vanadium dioxide which was doped with 0.5 wt % of tungsten exhibited a kind of an irregular shape morphology. However, by increasing the concentration of the dopant, SEM images of V_0.99_W_0.01_O_2_ and V_0.98_W_0.02_O_2_ shows the presence of a rod-like structure. This was attributed to the presence of VO_2_(A) by-product [26,31,32,33]. The above result is in a good agreement with the XRD results, indicated that the presence of VO_2_(A) by-product became more apparent at the high concentration of W dopant.

**Figure 6:**
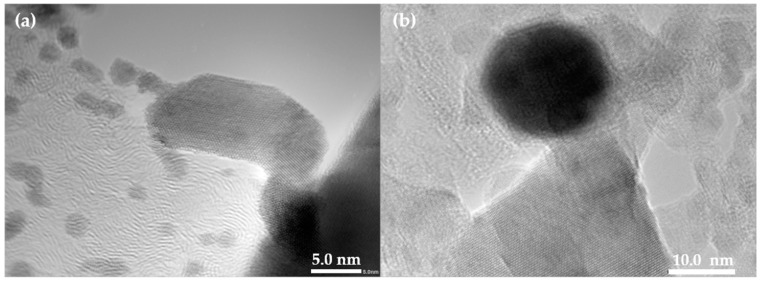
TEM images of the synthesized VO_2_ (**a**); and the doped VO_2_(M); V_0.995_W_0.005_O_2_ (**b**).

**Figure 7:**
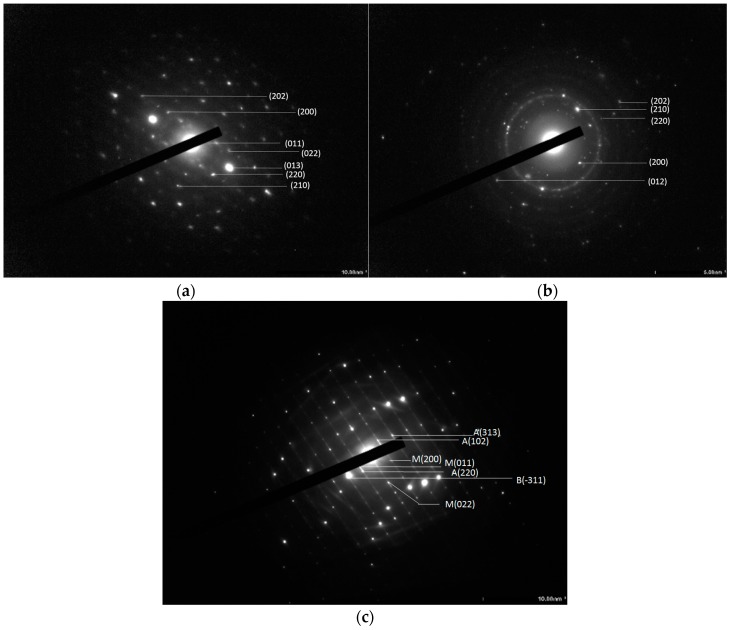
SAED patterns of VO_2_ (**a**); V_0.995_W_0.005_O_2_ (**b**); and V_0.98_W_0.02_O_2_ (**c**).

Figure 6 shows lattice fringe in the higher resolution TEM images of the normal VO_2_ and the doped VO_2_ (0.5 at % W). Granular shape particles were observed for both cases. This is consistent with that was observed from the SEM image. Size of the un-doped VO_2_ particles ranges 59 nm increased to 72 nm after doping. Similarly, Liu et al. [34] examined morphology of VO_2_/Si-Al gel by TEM and found that particle size of the composite was in the range of 20 nm. Attempts were also made to determine the d-spacing of the VO_2_, The results summarized in Table 1 shows that the values from both techniques are comparable, excepting the VO_2_ doped with 2 at % in which the lattice fringe in the TEM image was overlapped and unclear. In addition, SAED patterns of the VO_2_ (Figure 7) show the presence of various crystal planes, indicating that VO_2_ is polycrystalline. The similar patterns were observed from the 0.5 at % doped VO_2_. However, as the concentration of W dopant was further increased to 2 at %, the SAED pattern shows the presence of other planes corresponding to the additional VO_2_(B) and VO_2_(A) phases. The above results are in good agreement with those were obtained from the XRD patterns (Figure 2).

### 2.3. Thermal Behaviors

**Figure 8:**
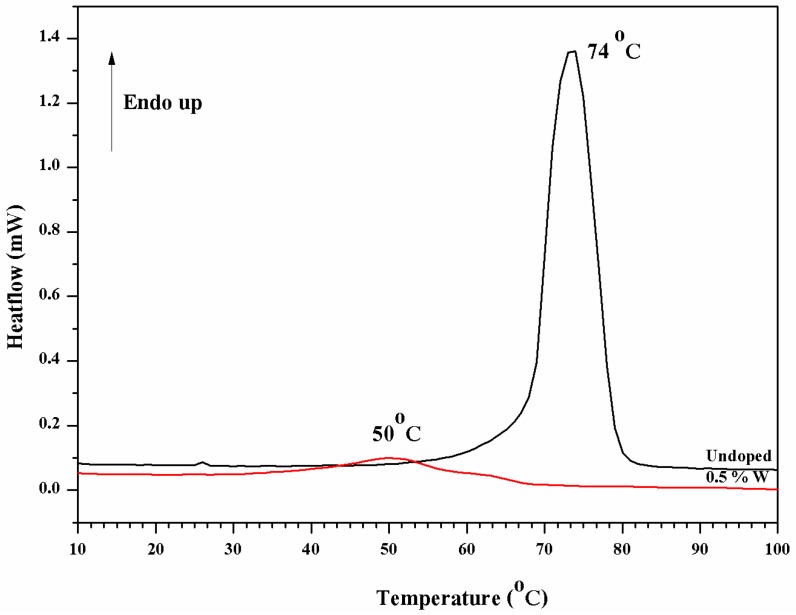
DSC thermograms of VO_2_ and the tungsten doped VO_2_.

Figure 8 shows DSC thermograms of VO_2_(M) both before and after doping. An exothermic peak at 74 °C can be seen from the thermogram of the un-doped VO_2_. This refers to phase transition temperature of the thermo-chromic material, changing from semiconductor (M) to metallic (R) structures. The exothermic peak, representing thermo-chromic transition of the metal oxide also was observed after doping it with 0.5 at % of tungsten. Noteworthy, the peak became broader after doping, due to a mal-distribution of factors, which caused the change in transition temperature [12,18]. Nevertheless, in this case, the peaks shifted downward from 74 °C to 50 °C. This indicates the tungsten dopant was capable of effectively lowering the transition temperature of the metal oxide. Wang et al. [4] studied doping effects of Mg/W in VO_2_ film and found the similar effect. For the VO_2_ doped with 2 at % of pure tungsten (without Mg co-dopant), it was found that transition temperature of the neat VO_2_ thin film decreased from 63.89 °C to a lower temperature (27.05 °C) as compared to our work. The discrepancy could be attributed to the fact that different percentage atomic of tungsten was used.

**Figure 9:**
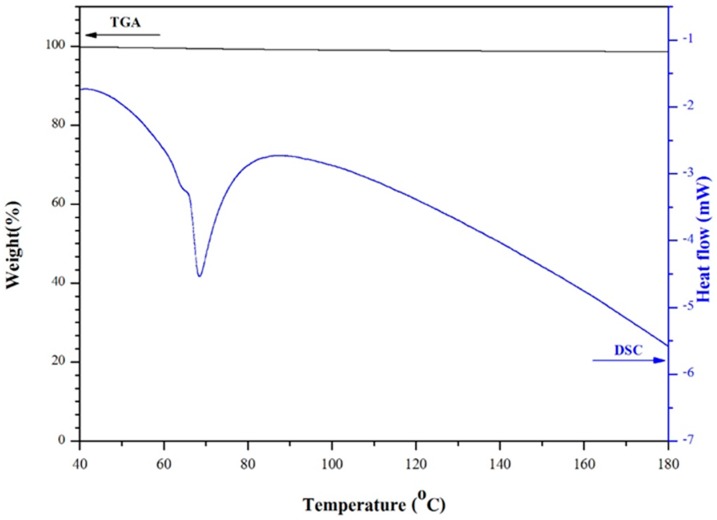
DSC-TGA thermograms of VO_2_(M).

Attempts were also made to follow up the enthalpy changes of VO_2_, concurrently with its thermal gravimetric analysis. From the DSC-TGA thermograms of the metal oxide (Figure 9), it can be seen that there was no weight loss occurred during the DSC transition at 68 °C. This indicates that the above transition was related to phase change of the thermo-chromic materials, and not due to the loss of any intermediate, residual or by-products.

### 2.4. Thermo-Chromic Behaviors

**Figure 10:**
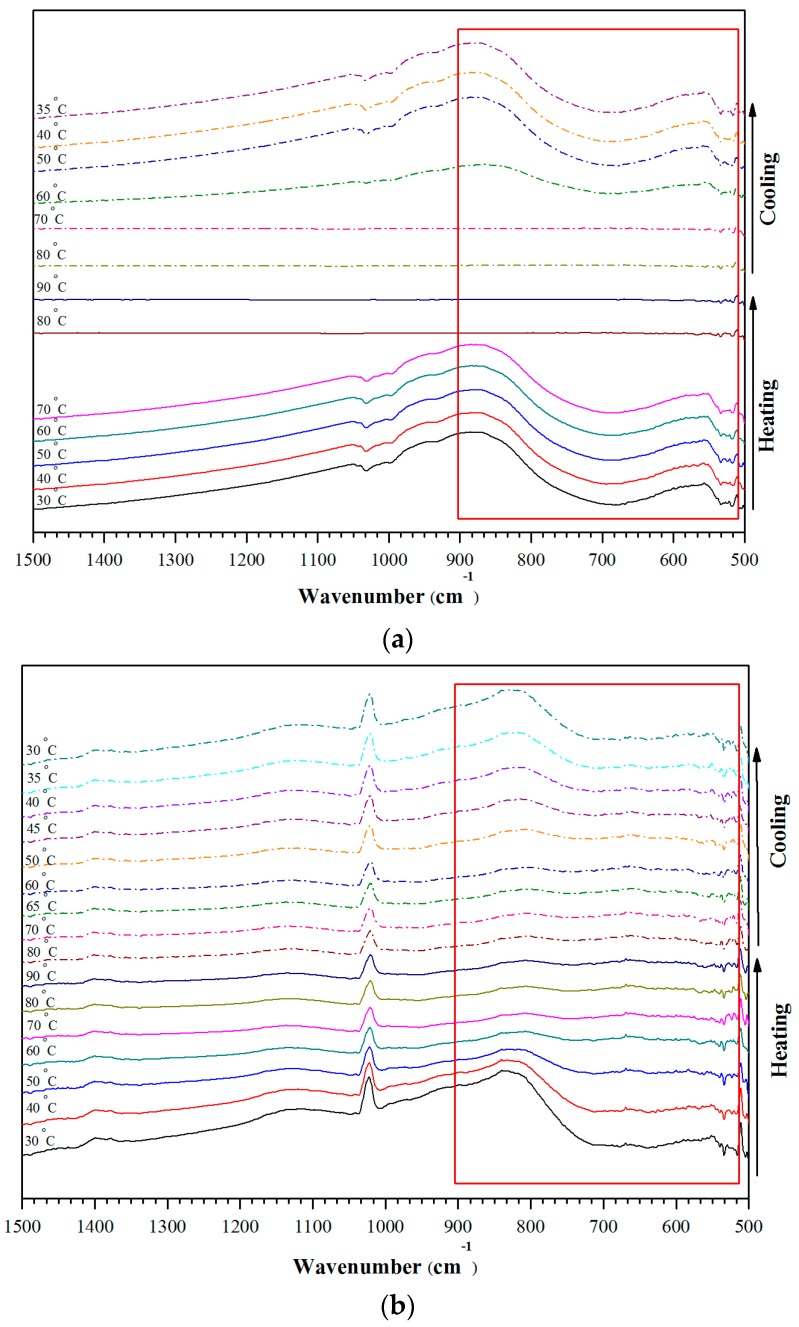
FT-IR spectra of the un-doped VO_2_(M) (**a**); and the doped VO_2_ (V_0.995_W_0.005_O_2_) (**b**), recorded as a function of temperature.

Figure 10 shows FTIR spectra of the VO_2_(M) particles, recorded as a function of temperature. It can be seen that absorbance of the broad peak over the wavenumber ranged between 500 and 900 cm^−1^, corresponding to the mid-IR region, remarkably changed as the running temperature increased. Specifically, when the scanning temperature was increased to 80 °C, the absorbance peak disappeared. This was due to the fact that the spectrum was recorded at a temperature above the phase transition temperature of the VO_2_(M) (74 °C, for the metal oxide without doping). This means that the VO_2_ had changed from monoclinic phase to rutile phase, accompanied with the change in optical properties from NIR transmittance to NIR reflectance. As the scanning temperature was cooled down from 90 °C toward the ambient temperature (35 °C), the peak emerged again. This indicates that thermo-chromic behavior of the materials is reversible. The similar behavior was observed from the FTIR spectra of the tungsten doped VO_2_. In this case, however, the absorbance peak disappeared at a lower temperature (60 °C) as compare to that of the normal VO_2_(M). This is due to the fact that phase transition temperature of the material dropped from 74 °C to 50 °C after doping. The above results are in good agreements with the results from XRD and DSC thermograms and are sufficient to confirm that the thermo-chromic VO_2_(M), with and without doping, were successfully prepared.

### 2.5. Structure-Properties of EVA/VO_2_ Composites

**Figure 11:**
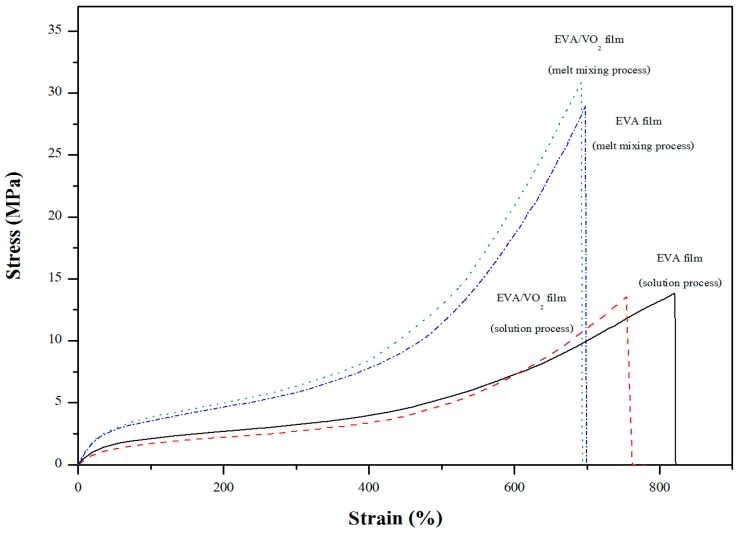
Stress-strain curves of EVA and EVA/VO_2_ composite films.

Figure 11 shows stress-strain curves of EVA and the EVA/VO_2_ composite films. Tensile properties of the various samples were also summarized in Table 2. The ultimate stress, strain at break and initial slope of the EVA hardly changed after applying 1 wt % of the VO_2_ particles into the polymer film. However, it was noted that the tensile properties significantly affected by the mixing process. The films prepared via the melt mixing process are stronger than those of which prepared by the solution mixing process. The discrepancies can be related to the lower gel content values of the solution casted films (Table 2) as compared to those of which prepared by the melt mixing process. This was, in turn, owing to some differences between the two processes, in terms of the actual curing conditions. Specifically, the melt mixed film was prepared by an internal mixer followed by curing in a hydraulic compression molding under high pressure. On the other hand, the solution casted film was mixed by solution before curing in a hot air oven without any pressure. In this regard, the shear rate, heat transfer and the actual temperature of the two processes could be different. These factors might promote the greater gel content and mechanical properties of the films prepared by the melt process.

**Figure 12:**
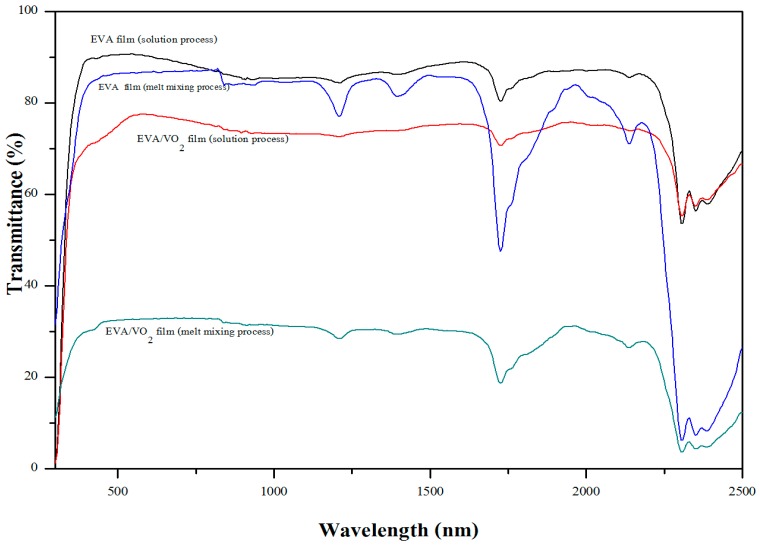
Transmittance spectra of EVA and EVA/VO_2_ composite films.

**Figure 13:**
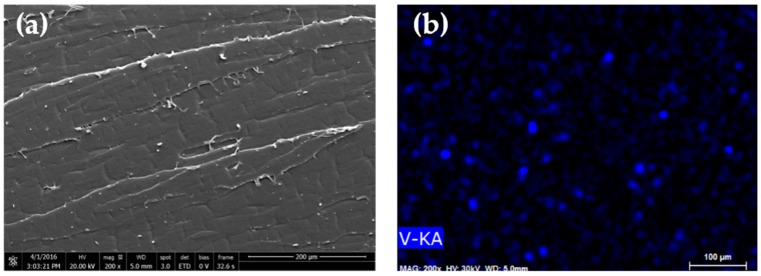

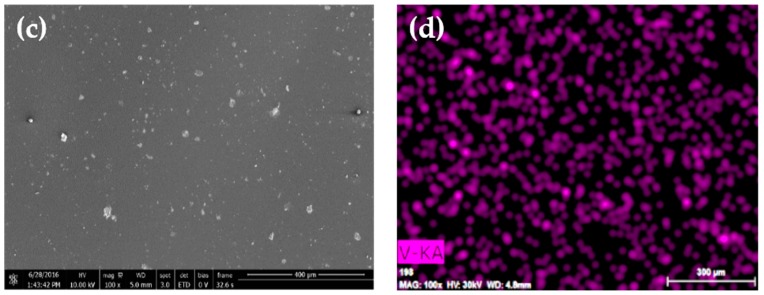
SEM images and X-ray dot map (V-Kα) of the EVA/VO_2_ prepared via melt blending (**a**,**b**); and those of the solution casted EVA/VO_2_ film (**c**,**d**).

**Figure 14:**
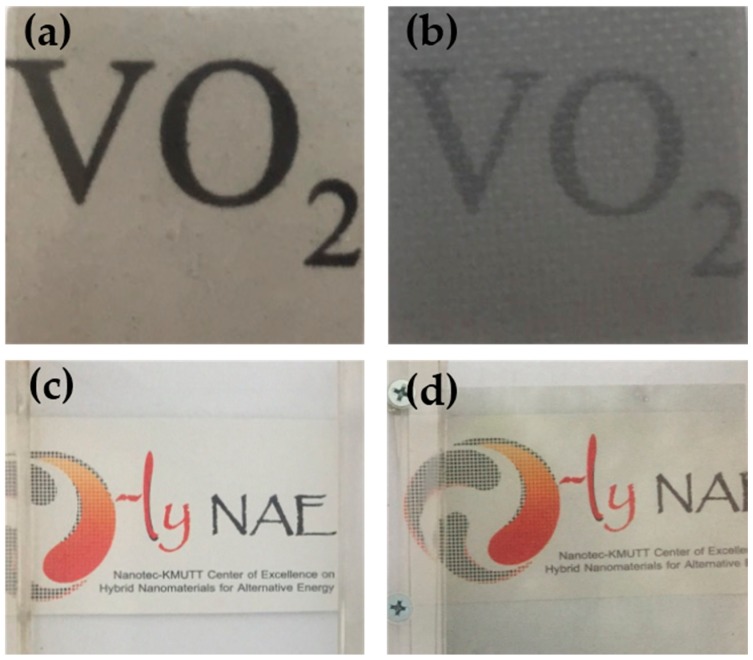
Photographs of the stand-alone solution casted EVA/VO_2_ film (**a**); the stand-alone melt mixed EVA/VO_2_ film (**b**); the EVA film coated glass (**c**); and the EVA/VO_2_ film coated glass (**d**).

Figure 12 shows the overlaid UV/Vis spectra of the various EVA films and the average visible light transmittance of the EVA films are summarized in Table 2. Regardless of the mixing processes, light transmittance in the visible range of the EVA was about 86%–89.95%. After mixing VO_2_ particles with EVA by a solution process, visible light transmittance of the solution casted film slightly decreased to 73.73%. This was due to the presence of the metal oxide particles which is inherently opaque. Particles size of the metal oxide, observed from the SEM images (Figure 13), is also considered large. Some of which are agglomerated in the polymer matrix. Nevertheless, the EVA/VO_2_ coated glass is still semi-transparent (see Figure 14). The effect of VO_2_ particles on visible light transparency of EVA film became more pronounced when the composite was prepared by melt mixing process. In this case, transmittance of the EVA/VO_2_ dropped rapidly as compared to that of the neat EVA film prepared by the same process. The EDX dot map of the specimens illustrated in Figure 13 showed that the VO_2_ particles are randomly distributed within the polymer matrix. However, size of the metal oxide particles is considerable. The VO_2_ particles are still agglomerated. It seems that, for the sake of a more desirable thermos-chromic/optical properties of the films, further work have yet to be carried out in order to improve dispersion of the VO_2_ particles in the polymer matrix. This can be achieved by several approaches including the adjustment of shear rate, mixing time, viscosity, and surface functionalization of the materials.

**Figure 15:**
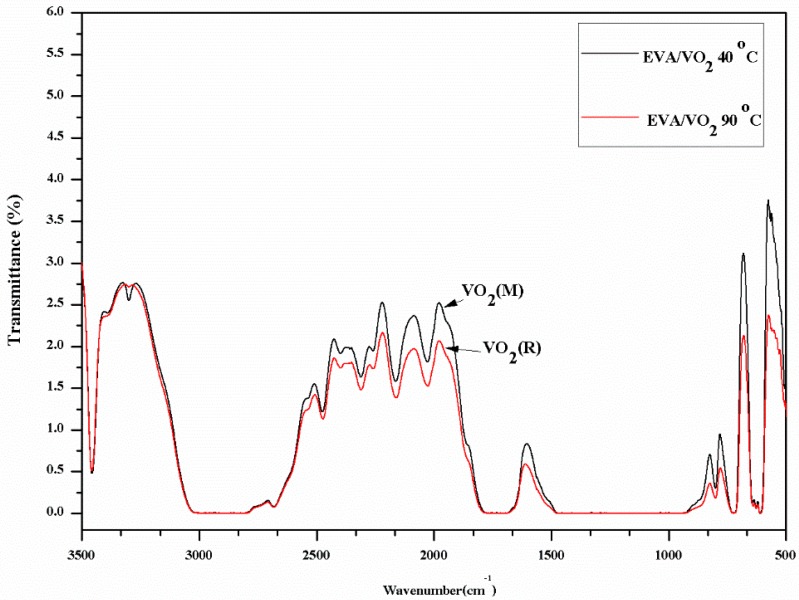
FTIR spectra of the solution casted EVA/VO_2_ (1 wt %) film recorded at 40 and 90 °C.

From the above results, it seems that the mixing process strongly affected mechanical, thermal and optical properties of the EVA/VO_2_ films. Percentage transmittance of the EVA/VO_2_ prepared via a solution mixing is greater than that of which prepared via the melt mixing process. The superior optical properties of the former were obtained at the expense of its tensile properties. In this study, visible light transparency and heat reflectance of the EVA/VO_2_ films are of higher priority taking into account its potential application as binder in laminated glass. Therefore, the composite film prepared via the solution mixing and casting processes was selected for a further study on thermo-chromic behaviors. Figure 15 shows the overlaid FTIR spectra of the EVA/VO_2_ composite films, which were recorded at two different temperatures. The FTIR transmittance over the wavenumber ranged between 500 and 3500 cm^−1^, corresponding to the mid IR region, of the films recorded at 90 °C, decreased as compared to that of which recorded at 40 °C. This implies that the EVA/VO_2_ film was capable of reflecting heat wave, provided that it was used at a temperature above the phase transition temperature of VO_2_(M). The similar results was observed Suzuki et al. [20] in the VO_2_-SiO_2_ particle/PLA composite, using the FTIR spectrophotometer to record the transmittance over the wavelength range between 2.5 µm and 8.5 µm (corresponding to the mid IR region). In that case, transmittance of the composite recorded at a high temperature (80 °C) was 10% lower than that of which recorded at a room temperature. However, PLA resin is inherently brittle, moisture sensitive, and expensive. In this regard, the application of VO_2_/PLA composite as coating for smart window might not be practical. It is also worth mentioning that changes in spectral transmittances of the VO_2_, recoded by FTIR, are less obvious as compared to those of which recorded by Zhou et al. [35]. In that case, solar modulation (∆T_sol_) of the VO_2_/hydrogel hybrid, recorded over the wavelength range from 500 to 2500 nm, as high as 34.7% was observed. Similarly, Shi et al. [19] found that NIR transmittance, at 2500 nm, of the acrylic resin/W doped VO_2_ coating decreased by up to 23% after increasing a temperature to above its transition.

**Figure 16:**
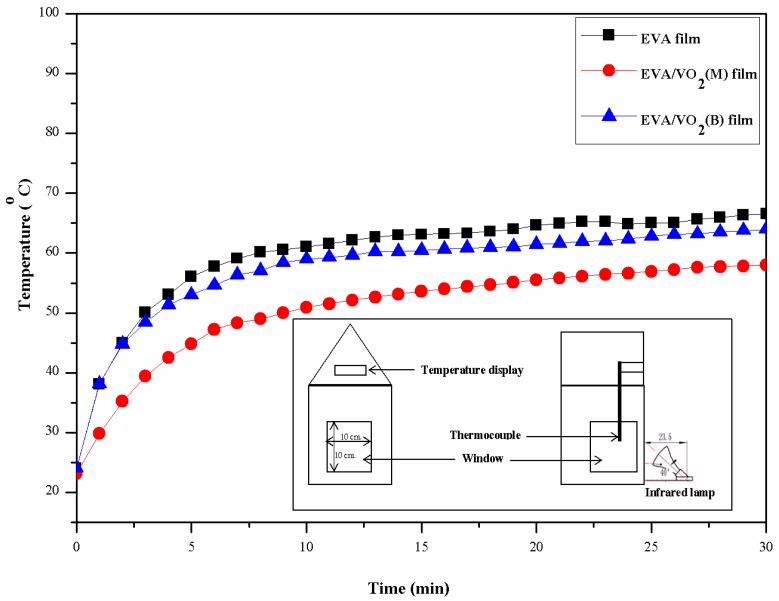
Changes in temperature inside the model house as a function of time irradiated with an IR lamp. The window was coated with EVA based films.

Last but not least, the above results were confirmed by considering the demo system, containing EVA and EVA/VO_2_ films coated on a window of a model house (see Figure 16; inset). An infrared lamp (PHILIPS, R125 IR R 150 W) was used as a heat source to activate phase change and thermo-chromic behavior of the VO_2_. The actual temperature in front of the window, measure by thermocouple was 100 ± 1 °C. This is well above the transition temperature of the VO_2_. Figure 16 shows changes in indoor temperature behind the windows as a function of irradiation time. It can be seen that the temperature linearly increased with time and reached a plateau after about 10 min. The equilibrium temperature behind the window coated with the normal EVA film was approximately 62 °C. The similar profiles were observed when the EVA film was replaced with either EVA/VO_2_(M) or EVA/VO_2_(B) composite films. Some discrepancies were noted, however, for these cases. The equilibrium temperature behind the window coated with EVA/VO_2_(M) was about 53 °C, which is significantly much lower than that of the control system (the use of a window with the normal EVA film). The above effect was not the case when EVA/VO_2_(B) film was used. Again, the difference can be ascribed to the fact that the VO_2_(M) is a kind of thermo-chromic material whereas the VO_2_(B) was not. This reflects that the main factor attributing to the decrease of temperature inside the model house is heat reflectance of the thermo-chromic VO_2_(M) particles, and not due to light scattering effect.

## 3. Materials and Methods

### 3.1. Materials

Vanadiam pentoxide (V_2_O_5_, >98% pure) was obtained from Sigma-Aldrich Co., Ltd. (St. Louis, MO, USA). Hydrazine monochloride (N_2_H_4_·HCl, analytically, >98% pure) was obtained from Acros organic Co., Ltd. (Morris Plains, NJ, USA). Hydrochloric acid (HCl, analytical pure) was obtained from Merck Co., Ltd. (Darmstadt, Germany). All of chemicals were used without further purification. EVA (Evaflex 150, containing 33 wt % vinyl acetate) was purchased from Mitsu-Dupont Co., Ltd. (Tokyo, Japan). Bis(2,2,6,6-tetramethyl-4-piperidinyl) sebacate (Tinuvin 770), used as a primary antioxidant, and 2,4-bis(1,1-dimethylethyl)phosphite (I) and dioctadecyl 3,30-thiopropionate (Irganox 802 FD), used as secondary antioxidants, were obtained from Ciba Specialty Co., Ltd. (Basel, Switzerland). The peroxide curing agent used in this study was a standard curing type, 2,5-bis(tert-butyldioxy)-2,5-dimethylhexane (Luperox 101), which was supplied by Arkema Co., Ltd. (Philadelphia, PA, USA). All chemicals were used as received.

### 3.2. Synthesis of VO_2_

The precursor solution of vanadyl dichloride (VOCl_2_) was prepared by gradually addition of 12 mL of a solution of hydrazine monochloride (1.67 M in HCl) into a suspension of 7 g of V_2_O_5_, in 100 mL of deionized water. After stirring for 24 h, the solution formed was filtered and a clear VOCl_2_ solution, in blue color with the pH value of about 1, was obtained. This VOCl_2_ precursor was then filled in a 250 mL Teflon tube before underwent a hydrothermal process in an autoclave at 200 °C for 5 h, 8 h, 12 h and 48 h. After that, the precipitate was filtered and washed with deionized water for three times, followed by washing with ethanol for three times. The purified precipitate was dried under vacuum at 80 °C for 3 h. Finally, it was calcined at 700 °C for 3 h. The similar procedures were used for preparing the tungsten doped VO_2_, using sodium tungstate as a dopant. In this case, 0.2, 0.4 and 0.8 mL of the dopant (0.1 M aqueous solution) was firstly dropped into the V_2_O_5_ suspension, followed by adding the solution of hydrazine (1.67 M, 12 mL). After that, the similar procedures were followed.

### 3.3. Preparation of EVA and EVA/VO_2_ Films

#### 3.3.1. Melt Mixing Process

EVA was compounded through the mixing of the polymer pellets with various additives using a compounding recipe illustrated in Table 3. The compounding was carried out in an internal mixer (LabTech Engineering Co., Ltd., Bangkok, Thailand). The mixing temperature, mixing time, rotor speed and the fill factor used were 120 °C, 15 min, 50 rpm, and 0.72, respectively. After that, the polymer was cooled and collected. Next, the EVA based films were fabricated with a hydraulic compression mold (LabTech Engineering Co., Ltd., Bangkok, Thailand) at 160 °C for a given time (t_90_ = 30 min). Noteworthy, before carrying out the compression molding, an oscillating disk rheometer (Gotech, Taipei, Taiwan) was used to determine the time to reach 90% of the maximum torque by the rheometer (t_90_) at 160 °C. This was used as the optimum cure time to vulcanize the EVA films. Thickness of the EVA based films prepared by compression molding process was 0.50 (±0.04) mm.

#### 3.3.2. Solution Mixing Process

Similar compounding recipes were used for preparing the EVA based films via a solution process. In this regard, the EVA solutions (10 wt %) were prepared by dissolving a given amount of EVA resin in 9 mL of chloroform, along with other chemicals as specified in Table 3. The solution was then kept stirring at room temperature for 3 h until its complete dissolution. After that, the polymer composite film was fabricated by pouring the solution onto a glass substrate (10 × 10 cm^2^). The casted film was dried at room temperature for 24 h or until reaching a constant weight. After that, the film was cured in a hot air oven, at 160 °C for 30 min. The measured thicknesses of the solution casted EVA and EVA/VO_2_ films were 0.590 (±0.04) and 0.587 (±0.03) mm, respectively.

### 3.4. Characterizations

The FTIR experiment was carried out in an attenuated FTIR (reflection) mode, using a Thermo instrument (iS5 model, Perkin Elmer, Spectrum one, Sacramento, CA, USA). The samples were scanned over wavenumbers ranging between 500 and 4000 cm^−1^ X-ray diffraction patterns of the synthesized VO_x_ were recorded by an X-ray diffractometer (XRD, AXS D8-Discover, Bruker, Karlsruhe, Germany) in the 2θ range of 10°–80° using Cu-Kα radiation (λ = 1.54178 Å). The accelerating voltage and the current used were 40 kV and 40 mA, respectively.

Morphology of the synthesized VO_2_ and the EVA composite films were examined using Scanning Electron Microscopy (SEM) technique. SEM experiment was operated using a JEOL (JSM 6610LV, JEOL, Peabody, MA, USA) machine, equipped with a secondary electron detector and energy dispersive X-ray detector (EDX). The accelerating voltages used was 10–30 kV. The sample was coated with gold prior to the SEM experiment in order to avoid charging effect during the electron beam scanning. The morphology and fringe pattern of the prepared particles were observed by high resolution transmission electron microscopy (HRTEM), using the JEOL JEM-2100 microscope (JEOL, Peabody, MA, USA) with an accelerating voltage of 200 kV. The TEM specimen was prepared by was dissolving 1 mg of the VO_2_ particle in 10 mL of DI water and was sonicated for 30 min. The solution was dropped on copper grid and was dried under room temperature. In addition to the TEM images, the attachment of selected area electron diffraction (SAED) of JEM-2100 was used to get the crystallographic information.

#### 3.4.1. Thermal Analysis

Phase transition temperatures of the synthesized VO_2_, with and without doping, as well as glass transition temperature (T_g_) and melt transition temperature values of the polymer composites were investigated by using a DSC technique. Typically, about 15 mg of the sample was used and the DSC experiment was carried out with a NETZSCH (DSC 204, NETZSCH, Watertown, MA, USA) instrument under a nitrogen atmosphere at a heating rate of 10 °C/min over temperatures ranging between 0 and 200 °C. Percentage crystallinity (X_c_) of the samples was calculated according to Equation (4):

(X_c_) = (*ΔHf*/ΔHf*) × 100 (4)

where ΔHf* is the enthalpy of fusion of the perfect polyethylene (PE) crystal; *ΔHf* is the enthalpy of fusion of the EVA samples, respectively. The value of ΔHf* for PE is 277.1 J/g [36].

In addition, the weight composition and thermal stability of the EVA composite films were concurrently determined along with the DSC experiment, using a DSC/thermal gravimetric analysis (TGA) techniques. In this regard, the DSC/TGA experiment was carried out with a NETZSCH (TGA 209 model). Approximately 8 mg of each sample was used and the TGA experiment was scanned over temperatures ranging between 25 °C and 900 °C under nitrogen gas and a heating rate of 10 °C/min.

#### 3.4.2. Testing of the EVA Based Films

The mechanical properties of the various EVA films were evaluated by tensile test, using Lloyd (LR 50 K, West Sussex, UK) instrument. Dumbbell-shaped specimens were prepared by cutting the dried films with a die, in accordance with the ASTM D638 standard. The gauge length used was 50 mm and the tensile test was carried out at a crosshead speed of 500 mm·min^−1^, using the 1 kN load cell. At least five specimens were tested for each sample and the average values of Young’s modulus, tensile strength at break, and elongation at break were calculated using standard equations. Tensile toughness was also calculated by using the area underneath the stress-strain curve.

Gel content of the cured EVA film was tested in accordance with the ASTM D-2765 standard method. About 1 g of the cured EVA films was immersed in xylene and then refluxed for 12 h. The specimens were then dried at 110 °C for 10 h before weighing. Gel content was evaluated using the following Equation (5):

Gel content = (W_1_/W_2_) × 100 (5)

where W_1_ = the swollen weight of the specimen after an immersion in xylene; W_2_ = the dried weight of the specimen.

UV/Visible absorption spectra of various samples were recorded on a Shimadzu UV-3100 spectrophotometer (Shimadzu, Tokyo, Japan) over wavelengths ranging between 200 and 1000 nm. Visible light transmittance was determined in accordance with ISO 9050 standard method. Specifically, transmission of light through the polymer film was integrated over the wavelength range of 400–700 nm. Total reflectance measurements were obtained in the solar spectrum from 300 to 2500 nm at an incident angle of 15 degrees. The spectral data was integrated against Air Mass 1.5 global spectrum (ASTM E891) to yield weighted ordinates over the total spectral bandwidth. Five measurements were made of each sample and the weight averaged values were reported.

## 4. Conclusions

Monoclinic VO_2_ particles, with and without doping were successfully prepared. The results were confirmed by XRD and DSC techniques. Thermo-chromic behaviors of the metal oxides were demonstrated by the changes in FTIR absorbance as a function of temperature. After mixing the VO_2_(M) particles with EVA, thermo-chromic and heat reflectance behaviors of the composite materials still exist. The presence of VO_2_ (1 wt %) in EVA did not significantly affected physical and thermo-mechanical properties of the polymer films, regardless of the mixing processes used. On the other hand, type and conditions of the mixing processes strongly affected mechanical, thermal and optical properties of the EVA/VO_2_ films. The results summarized in Table 3 shows that tensile strength and modulus of the EVA based films prepared via the melt process was greater than those of which prepared by a solution process. Better properties of the former system were obtained at the expense of its percentage light transmittance. For the sake of better optical and thermo-chromic properties of the EVA/VO_2_ films, dispersion of the metal oxide in the polymer matrix has yet to be further improved. The optimum concentration of VO_2_ in the EVA based film also need to be investigated.

## Figures and Tables

**Table 1:** Inter-planar distance or d-spacing of the (011) plane and size of the VO_2_(M) and (V_1−*x*_W*_x_*O_2_) crystals which were prepared by using various concentration of tungsten dopant.

Tungsten (% Atom)	d-Spacing (nm)		Crystal Size (nm)
	From XRD	From TEM	
0	0.3199	0.3550	26.9
0.5	0.3209	0.3586	22.2
1.0	0.3202	n/a	25.6
2.0	0.3209	0.2460	28.1

**Table 2:** Tensile, physical and optical properties of EVA and EVA/VO_2_ composite films.

Properties	EVA Films		EVA/VO_2_ (1 wt %) Films	
	Melt Mixing	Solution Mixing	Melt Mixing	Solution Mixing
Modulus (MPa)	14.29 (±0.99)	4.70 (±0.73)	13.12 (±1.23)	5.31 (±0.34)
Ultimate tensile Stress (MPa)	29.75 (±3.75)	13.63 (±2.00)	32.25 (±1.39)	13.89 (±0.76)
Strain (%)	604 (±37)	802 (±17.81)	696 (±9)	745 (±14.72)
Toughness (J)	6.20 (±0.87)	7.14 (±0.59)	7.58 (±0.39)	6.10 (±0.49)
Gel content (%)	83.62 (±3.49)	45.90 (±1.11)	93.25 (±1.89)	34.80 (±2.20)
Visible light transmittance (%)	85.98 (±0.97)	89.95 (±0.54)	31.60 (±0.73)	73.73 (±0.56)

**Table 3:** Compounding formulations of EVA composite films.

Chemicals (Trade Names)	Formulation/Content (phr)	
	EVA-1	EVA-2
EVA Polymer (Evaflex 150)	100	100
Primary Antioxidant (Tinnuvin 770)	0.1	0.1
Secondary Antioxidant (Irganox PS 802FD)	0.2	0.2
Peroxide Curing Agent (Luperox 101)	1.5	1.5
VO_2_	0	1
